# circRNA6448-14/miR-455-3p/OTUB2 axis stimulates glycolysis and stemness of esophageal squamous cell carcinoma

**DOI:** 10.18632/aging.205879

**Published:** 2024-05-30

**Authors:** Yaowen Zhang, Heming Zhang, Chenyu Wang, Shasha Cao, Xinyu Cheng, Linzhi Jin, Runchuan Ren, Fuyou Zhou

**Affiliations:** 1 Department of Radiation Oncology, Anyang Tumor Hospital, The Affiliated Anyang Tumor Hospital of Henan University of Science and Technology, Henan Medical key Laboratory of Precise Prevention and Treatment of Esophageal Cancer, Anyang 455000, China

**Keywords:** circRNA6448-14, miR-455-3p, OTUB2, esophageal squamous cell carcinoma

## Abstract

Background: Esophageal squamous cell carcinoma (ESCC) is a gastrointestinal malignancy with high incidence. This study aimed to reveal the complete circRNA-miRNA-mRNA regulatory network in ESCC and validate its function mechanism.

Method: Expression of OTU Domain-Containing Ubiquitin Aldehyde-Binding Protein 2 (OTUB2) in ESCC was analyzed by bioinformatics to find the binding sites between circRNA6448-14 and miR-455-3p, as well as miR-455-3p and OTUB2. The binding relationships were verified by RNA Immunoprecipitation (RIP) and dual-luciferase assay. The expressions of circRNA6448-14, miR-455-3p, and OTUB2 were detected by quantitative real-time polymerase chain reaction (qRT-PCR). MTT assay measured cell viability, and the spheroid formation assay assessed the ability of stem cell sphere formation. Western blot (WB) determined the expression of marker proteins of stem cell surface and rate-limiting enzyme of glycolysis. The Seahorse XFe96 extracellular flux analyzer measured the rate of extracellular acidification rate and cellular oxygen consumption. Corresponding assay kits assessed cellular glucose consumption, lactate production, and adenosine triphosphate (ATP) generation.

Results: In ESCC, circRNA6448-14 and OTUB2 were highly expressed in contrast to miR-455-3p. Knocking down circRNA6448-14 could prevent the glycolysis and stemness of ESCC cells. Additionally, circRNA6448-14 enhanced the expression of OTUB2 by sponging miR-455-3p. Overexpression of OTUB2 or silencing miR-455-3p reversed the inhibitory effect of knockdown of circRNA6448-14 on ESCC glycolysis and stemness.

Conclusion: This research demonstrated that the circRNA6448-14/miR-455-3p/OTUB2 axis induced the glycolysis and stemness of ESCC cells. Our study revealed a novel function of circRNA6448-14, which may serve as a potential therapeutic target for ESCC.

## INTRODUCTION

Esophageal cancer (EC) ranks eighth globally in terms of incidence and is the sixth leading cause of cancer-related deaths [1]. According to statistics, there were over 600,000 new cases and 540,000 deaths worldwide in 2020. Esophageal squamous cell carcinoma (ESCC) is one of the primary histological subtypes of EC, accounting for 85% of EC cases in the world [2]. The high-risk EC regions of Asia (including East, Central, and South Asia) and Africa (including East and Southern Africa) accounted for about 80% of EC cases, primarily ESCC [1, 3, 4]. The majority of ESCC patients received their diagnosis when the disease was either in the middle or late stages and there was no longer any hope for recovery. In certain countries, the 5-year survival rate is as low as 5%, with less than 20% overall [5, 6]. It has been shown that cancer stem cells (CSCs), a subpopulation of cells with self-renewal, multidirectional differentiation potential, and tumorigenic ability, are the underlying cause of tumorigenesis, progression, recurrence, metastasis, and drug resistance [7]. It is now well established that the poor prognosis of ESCC patients is closely associated with CSCs [8]. Clinical studies have eliminated CSCs by targeting Wnt, NOTCH, Hedgehog, and Hippo signaling pathways [9]. However, there is less consensus on biomarkers for CSCs in ESCC. Therefore, investigating the pathogenic mechanisms of ESCC and looking for more trustworthy targets is imperative considering these situations.

Circular RNA (circRNA) was originally thought to be a byproduct of erroneous splicing, formed by reverse splicing of pre-mRNA into a single-stranded covalently closed RNA molecule [10], and it was resistant to RNA nucleases due to its closed loop structure, which increased the stability and reduced the susceptibility to degradation of their expression [11]. Researchers have recently started to focus on the role of circRNA in various human diseases, including cancer [12–14]. In cancers, the crucial roles of circRNA have been confirmed in gastric cancer [15], colorectal cancer [16], lung cancer [17], breast cancer [18], etc. In certain cases, circRNA functions as a transcription factor, miRNA sponge, protein scaffold, or even a protein translation template [19]. Research on ESCC has focused on the dysregulation of circRNA as a diagnostic biomarker [20] and its role in disease progression [21–23], while the circRNA-miRNA-mRNA regulatory network awaits further exploration. Shen et al. [24] constructed a circRNA-miRNA-mRNA interaction network based on ESCC microarray profiles and public databases, and then identified hub genes, validating the significant function of the circRNA-miRNA-mRNA regulatory network in ESCC. Recently, the studies by Wang et al. [25], Zhou et al. [26], and Song et al. [27] enriched the circRNA-miRNA-mRNA regulatory network in ESCC to some extent. Regrettably, a deeper comprehension of the regulatory mechanisms had not been advanced by these studies. Previous research revealed that ESCC patients with high expression of circRNA6448-14 have poor prognosis, poor differentiation, and high pTNM stage. Cell progression is accelerated by circRNA6448-14. Simultaneously, circRNA6448-14 can act as a sponge for miR-455-3p [28]. This study aimed to define the circRNA-miRNA-mRNA regulatory network and to complete the precise mechanism of this regulatory axis in ESCC progression by investigating the downstream target genes of miR-455-3p.

In this study, we explored the effects of circRNA6448-14 on glycolysis and cell stemness in ESCC cells and, based on this, further explored its downstream molecular regulatory mechanisms. In conclusion, our research added to the understanding of the circRNA-miRNA-mRNA regulatory network in ESCC and offered a novel and solid clue for its treatment.

## MATERIALS AND METHODS

### Bioinformatics analysis

We obtained the expression of circRNA6448-14 and miR-455-3p in ESCC tissues from previous studies and verified that circRNA6448-14 could sponge miR-455-3p [28]. After using miRwalk to find the downstream target genes of miR-455-3p and performing a literature review, OTU Domain-Containing Ubiquitin Aldehyde-Binding Protein 2 (OTUB2) was ultimately determined to be the target. OTUB2 differential expression in ESCC was analyzed based on The Cancer Genome Atlas (TCGA)-ESCC-mRNA expression data (normal: 3, tumor: 96).

### Cell cultivation

All cells used in this study, human embryonic kidney cells (293T), normal human esophageal epithelial cells (HEEC), and ESCC cell lines (TE1, Eca109, KYSE150) were purchased from BeNa Culture Collection (BNCC, China) and cultured according to the recommended protocol. 293T cells were cultivated in Dulbecco’s modified Eagle’s medium-high glucose (DMEM-H), HEEC cells were cultivated in Eagle’s minimal essential medium (EMEM), TE1 and Eca109 cells were cultivated in RPMI-1640 medium, KYSE-150 cells were cultured in KYSE-150 cell special medium (45% RPMI-1640 + 45% F-12 + 10% fetal bovine serum (FBS)), all media contained 1% penicillin-streptomycin mixture and 10% FBS, and the DMEM-H medium for 293T cells also contained 2 mM L-glutamine [29]. All cells were cultured in the incubator at 37°C with 5% CO_2_. The media were purchased from BNCC (China), and the reagents were Gibco products from Thermo Fisher Scientific (USA).

### Cell transfection

siRNA targeting circRNA6448-14, miR-455-3p inhibitor, miR-455-3p mimic, OTUB2 overexpression vector, and corresponding controls were synthesized by RiboBio (China). According to instructions, the above vectors (1 μg/mL) were transfected into ESCC cells or 293T cells utilizing Lipofectamine™ 2000 (Invitrogen, USA), and the transfection efficiency was detected after 48 hours [30].

### qRT-PCR

Total RNA was isolated from cells using RNAsimple Total RNA Kit (TIANGEN, China), and reverse transcription was performed to obtain cDNA using Quantitect Reverse Transcription Kit (Qiagen, Germany). Next, Power Green qPCR Mix (Takara Bio, Japan) used the Applied Biosystems™ 7500 real-time PCR system to conduct the qRT-PCR assay. Finally, relative expression levels were calculated using 2^−ΔΔCt^ with GAPDH or U6 as controls [31]. The primer sequences are in the Table 1.

**Table 1:** qRT-PCR primer sequences.

**Gene**	**Sequence**
hsa_circ RNA6448-14	Forward: 5′-CCAATGGGGACTGTCATGGA-3′
	Reverse: 5′-TCATGCCGTGTTTCAGCTCA-3′
miR-455-3p	Forward: 5′-CTCAACTGGTGTCGTGGAGTCGGCAATTCAGTTGAGGTGTAT-3′
	Reverse: 5′-ACACTCCAGCTGGGGCAGTCCATGGGCAT-3′
U6	Forward: 5′-AACGCTTCACGAATTTGCGT-3′
	Reverse: 5′-CTCGCTTCGGCAGCACA-3′
GAPDH	Forward: 5′-GAAGGTGAAGGTCGGAGTC-3′
	Reverse: 5′-GAAGATGGTGATGGGATTTC-3′

### Western blot (WB)

The collected cells were lysed utilizing radioimmunoprecipitation assay (RIPA) buffer (Beyotime, China) for 30 minutes to obtain the total protein. The bicinchoninic acid (BCA) assay kit (Beyotime, China) was utilized to quantify the protein concentration. The membrane was transferred after SDS-PAGE electrophoresis. Then, the membrane was blocked with 5% BCA for 2 hours and incubated at 4°C overnight with primary rabbit antibodies (Abcam, UK): anti-Nanog (ab109250, 1:1000), anti-OCT4 (ab181557, 1:1000), anti-SOX2 (ab92494, 1:1000), anti-HK2 (ab209847, 1:1000), and anti-LDH4 (ab52488, 1:5000). The next day, after washing with TBST (5 min × 3), the membrane was incubated for 2 hours in the secondary antibody, goat anti-rabbit Immunoglobulin G (IgG) H&L (HRP) (ab6721, 1:2000, Abcam, UK). Finally, Western Blotting Luminol Reagent (Santa Cruz, USA) was used to achieve visualization in the ChemiScope6000 system [32].

### MTT assay

The transfected cells were seeded into 96-well plates at 2000 cells per well and cultured until the cells adhered. After 0, 24, 48, and 72 hours of cultivation, each well was added with 20 μl MTT solution (5 mg/mL). After 4 hours of incubation, 150 μl of DMSO was added and shaken for 10 minutes. A microplate reader (MTT and DMSO purchased from Solarbio, China) was used to measure the absorbance at 570 nm wavelength [33].

### Stem cell sphere formation assay

6-well ultra-low attachment plates (Corning, USA) were seeded with 4 × 10^4^ cells per well. The DMEM/F-12 medium was used to cultivate the cells, and it was supplemented with β-FGF (10 ng/mL), B27 (20 ng/mL), IGF (20 ng/mL), and human EGF (20 ng/mL). Cells were counted and photographed under an inverted microscope after 14 days of cultivation at 37°C with 5% CO_2_ [34]. Reagents and culture media were from Invitrogen (USA).

### Extracellular acidification rate (ECAR) and oxygen consumption rate (OCR) detection

The Seahorse XFe96 extracellular flux analyzer (Seahorse Bioscience, USA) was used to measure the ECAR and OCR. After cells were harvested and counted, 1 × 10^4^ cells/well were seeded into a SeahorseXF96 cell culture microplate and cultured for 10 hours. Afterwards, the Seahorse XF glycolysis Stress Test Kit and Seahorse XF Cell Mito Stress Test Kit (Agilent, USA) were used to measure ECAR and OCR, respectively. Glucose, and oligomycin or 2-DG should be added at specified times in the determination of ECAR. Oligomycin, and FCCP or antimycin A and Rotenone should be added at specified times in the determination of OCR [35].

### Lactate production, glucose consumption, and ATP detection

The Lactate Assay Kit, Glucose Uptake Assay Kit, and Luminescent ATP Detection Assay Kit (Solarbio, China) were used to detect lactate production, glucose consumption, and ATP generation, respectively.

### Dual-luciferase reporter assay

First, we constructed pmirGLO-circRNA6448-14-WT (wild-type), pmirGLO-circRNA6448-14-MUT (mutant-type), pmirGLO-OTUB2-MUT, and pmirGLO-OTUB2-MUT plasmids. The 3′UTR regions containing the binding sites of miR-149-3p in circRNA6448-14 or OTUB2 fragments, as well as the 3′UTR regions of circRNA6448-14 or OTUB2 fragments with mutated binding sites, were inserted into the pmirGLO vector. Plasmid and miR-455-3p mimic or NC mimic were co-transfected into 293T cells. As directed by the manufacturer, the Dual-Luciferase Reporter Assay System (Promega, USA) was utilized to measure the luciferase activity.

### Statistical analysis

Data were displayed as mean ± standard deviation of a minimum of three independent experiments. The Student’s *t*-test was used to compare the differences between the two groups. Groups with continuous variables were compared using analysis of variance. *P* < 0.05 implied a significant difference.

### Data availability statement

The data and materials in the current study are available from the corresponding author on reasonable request.

## RESULTS

### circRNA6448-14 is highly expressed in ESCC

**Figure 1:**
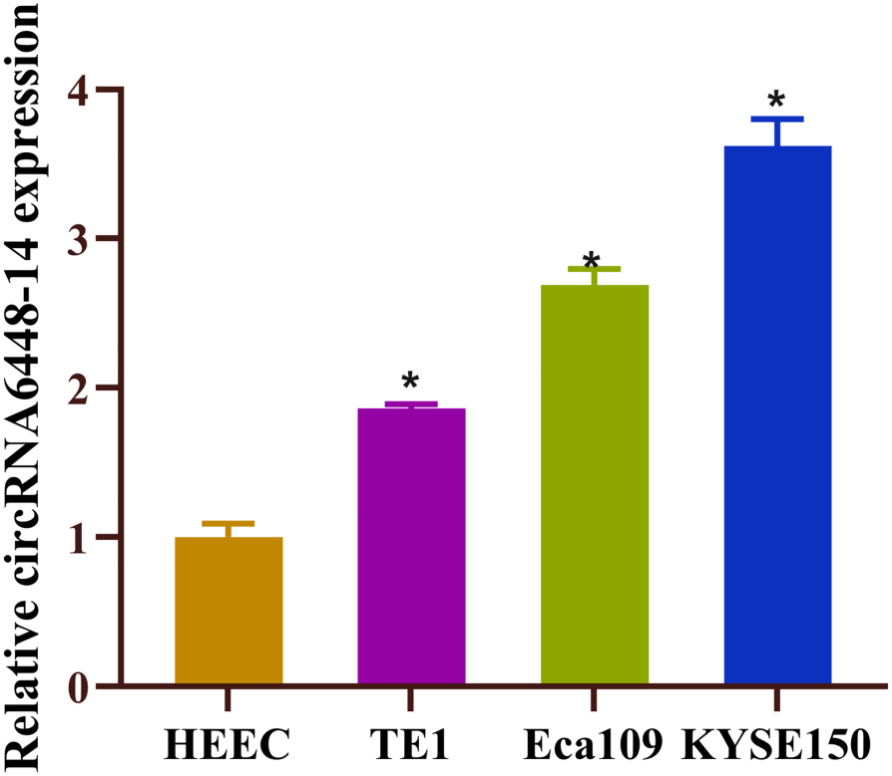
**High expression of circRNA6448-14 in ESCC.** Expression of circRNA6448-14 in normal esophageal epithelial cell HEEC and ESCC cells TE1, Eca109, KYSE150. ^*^P < 0.05.

In previous study, we investigated the expression profiles of circRNAs in ESCC and adjacent normal tissues by microarray analysis and selected upregulated circRNA, circRNA6448-14, based on the following criteria: (1) fold change, *P*-value; (2) the number of miRNA binding sites; (3) presence of target miRNAs associated with ESCC. It has been established that ESCC tissues express circRNA6448-14 at a higher level than normal tissues [28]. This finding led us to investigate the expression level of circRNA6448-14 in ESCC cell lines (TE1, Eca109, KYSE150), and the results suggested that these cell lines also had high expression levels of circRNA6448-14 (Figure 1). In conclusion, circRNA6448-14 was highly expressed in ESCC.

### circRNA6448-14 stimulates ESCC glycolysis and stemness

**Figure 2:**
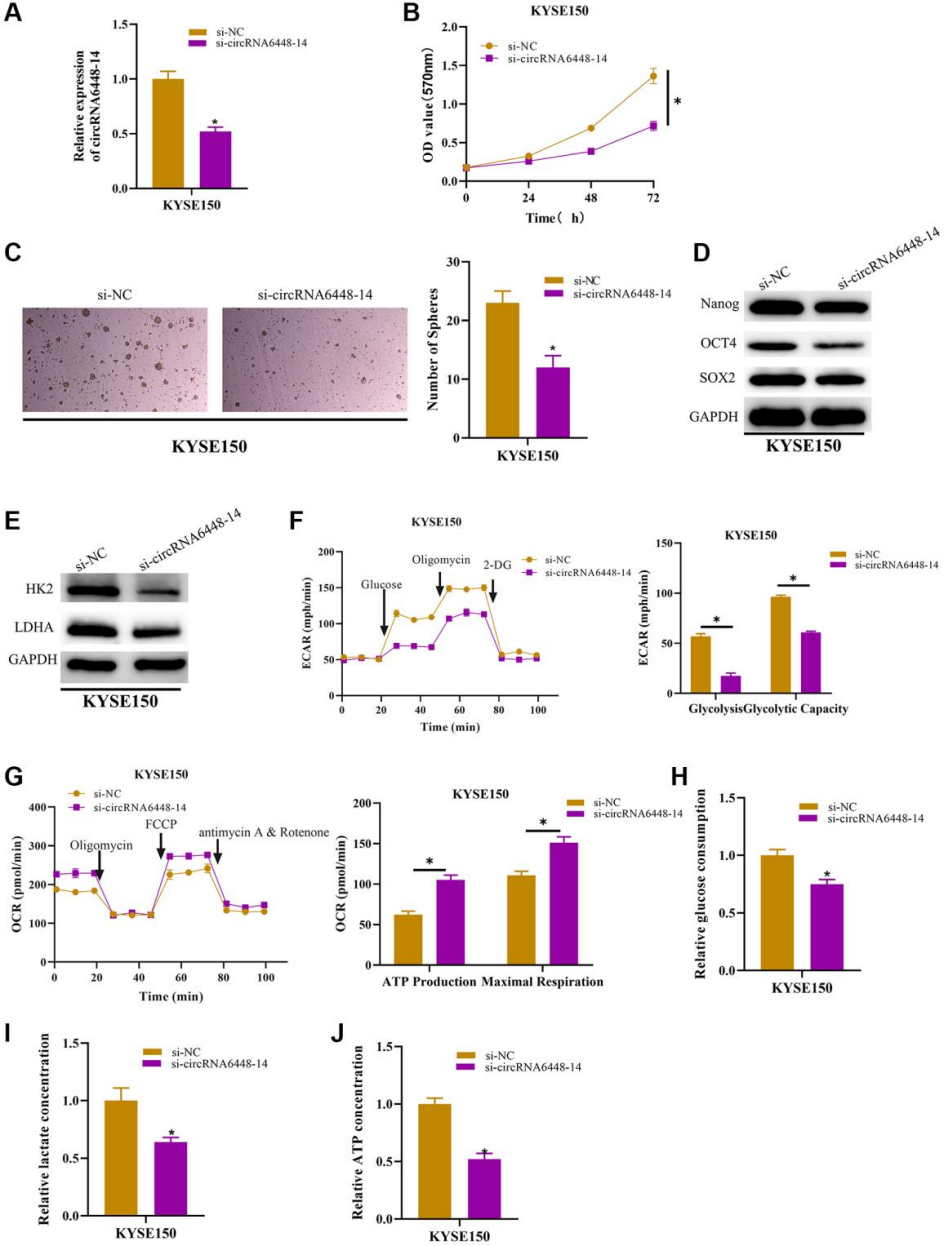
**circRNA6448-14 increases stemness and glycolysis in ESCC cells.** (**A**) qRT-PCR detection of circRNA6448-14 expression in circRNA6448-14 knockdown and control cells. (**B**) MTT assay detection of cell viability in circRNA6448-14 knockdown and control cells. (**C**) Stem cell sphere formation experiment in circRNA6448-14 knockdown and control cells. (**D**, **E**) WB detection of stem cell surface marker proteins Nanog, OCT4, SOX2 expression and glycolysis rate-limiting enzymes HK2, LDHA expression in circRNA6448-14 knockdown and control cells. (**F**) The ECAR of circRNA6448-14 knockdown and control cells. (**G**) The OCR of circRNA6448-14 knockdown and control cells. (**H**–**J**) The kits detected glucose consumption, lactate production, and ATP generation levels in cells. ^*^P < 0.05.

It was determined that circRNA6448-14 enhanced the capacity of ESCC cells to migrate and invade and circRNA6448-14 could be enriched in the pathways related to the regulation of tumor protein polysaccharide, according to previous studies [28]. Therefore, we speculated that circRNA6448-14 may stimulate the glycolysis and stemness of ESCC cells. We carried out several experiments to verify this hypothesis. First, si-NC and si-circRNA6448-14 cell groups were constructed, and it was proved that circRNA6448-14 expression was notably reduced in the circRNA6448-14 knockdown group (Figure 2A). MTT assay indicated the inhibitory effect of circRNA6448-14 knockdown for cell viability (Figure 2B). KYSE150 sphere formation ability was markedly suppressed by circRNA6448-14 knockdown in the stem cell sphere formation assay (Figure 2C). After determining the expression of glycolysis rate-limiting enzymes (HK2, LDHA) and stem cell surface markers (Nanog, OCT4, SOX2) in KYSE150 cells with circRNA6448-14 knockdown, it was discovered that these expressions were dramatically lower (Figure 2D, 2E). The circRNA6448-14 knockdown group demonstrated a notable increase in OCR detection and a major decrease in ECAR detection when we performed ECAR and OCR assays (Figure 2F, 2G). The results reflected that knockdown of circrna6448-14 decreased overall glycolytic flux as well as enhanced mitochondrial oxidative respiration in KYSE150 cells. Finally, we determined the effect of circRNA6448-14 knockdown on glycolytic phenotypes including lactic acid production, glucose consumption, and ATP production in KYSE150 cells. We found that knocking down circRNA6448-14 inhibited lactate production, glucose consumption, and ATP production in KYSE150 cells (Figure 2H–2J). In conclusion, circRNA6448-14 promoted ESCC cell proliferation in glycolysis and stemness.

### Functional relationship of circRNA6448-14/miR-455-3p/OTUB2 axis

**Figure 3:**
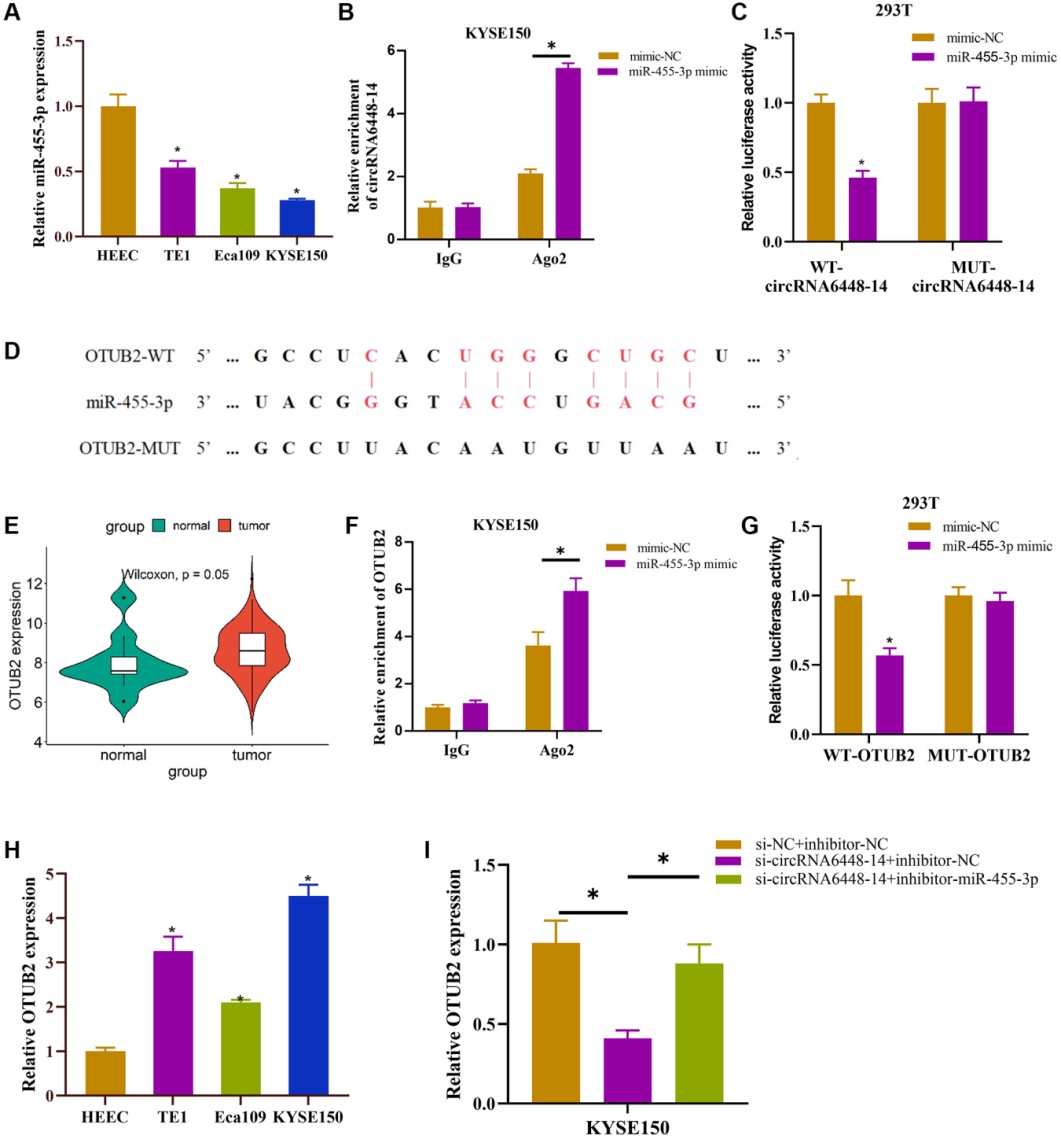
**Functional relationship of circRNA6448-14/miR-455-3p/OTUB2 axis.** (**A**) qRT-PCR detection of miR-455-3p expression in normal esophageal epithelial cell HEEC and ESCC cells TE1, Eca109, KYSE150. (**B**, **C**) RIP assay and dual luciferase assay were used to verify the binding relationship between circRNA6448-14 and miR-455-3p. (**D**) Binding site of miR-455-3p and OTUB2. (**E**) Expression of OTUB2 in ESCC and normal tissues. (**F**, **G**) RIP assay and dual luciferase assay were used to verify the binding relationship between miR-455-3p and OTUB2. (**H**) qRT-PCR detection of OTUB2 expression in normal esophageal epithelial cells HEEC and ESCC cells TE1, Eca109, KYSE150. (**I**) qRT-PCR detection of OTUB2 expression in different treatment groups. ^*^P < 0.05.

Recent research suggested that many circRNAs may act as miRNA sponges [36]. The previous study had constructed the circRNA6448-14-miRNA network and demonstrated that circRNA6448-14 could sponge miR-455-3p [28]. miR-455-3p expression was identified and shown to be downregulated in ESCC cell lines (Figure 3A). Furthermore, we performed RIP and dual luciferase assay to validate the binding relationship between circRNA6448-14 and miR-455-3p. According to the RIP results, the group treated with miR-455-3p mimic exhibited significantly higher levels of co-precipitation of AGO2 and circRNA6448-14 in comparison to the IgG group (Figure 3B). According to the dual luciferase assay, transfection of mimic-miR-455-3p significantly reduced the luciferase activity of the WT-circRNA6448-14 group but had no obvious effect on the MUT-circRNA6448-14 group (Figure 3C). Moreover, we used miRwalk to forecast the downstream mRNA regulated by miR-455-3p to enhance the circRNA-miRNA-mRNA network, and ultimately, we chose OTUB2 as the target (binding site shown in Figure 3D). The expression analysis of OTUB2 in TCGA-ESCC-mRNA data revealed its high expression in ESCC tissues (Figure 3E). According to RIP experiments, the group treated with miR-455-3p mimic had a fairly high level of AGO2 enrichment when co-precipitating with OTUB2 than the IgG group (Figure 3F). Meanwhile, in the dual luciferase assay, the WT-OTUB2 group treated with miR-455-3p mimic showed a great decrease in luciferase activity, whereas the MUT-OTUB2 group did not show any huge change (Figure 3G). We discovered that OTUB2 displayed an upregulated trend in ESCC cell lines (Figure 3H). Finally, to find out the relationship between circRNA6448-14/miR-455-3p/OTUB2 axis, we set up si-NC+inhibitor-NC, si-circRNA6448-14+inhibitor-NC, and si-circRNA6448-14+inhibitor-miR-455-3p groups and detected the expression of OTUB2 in each group. It was revealed that OTUB2 expression was inhibited when circRNA6448-14 was knocked down alone but was restored when adding miR-455-3p inhibitor (Figure 3I). In conclusion, circRNA6448-14 promoted the expression of OTUB2 by sponge adsorption of miR-455-3p.

### circRNA6448-14/miR-455-3p/OTUB2 axis promotes ESCC cells glycolysis and stemness

**Figure 4:**
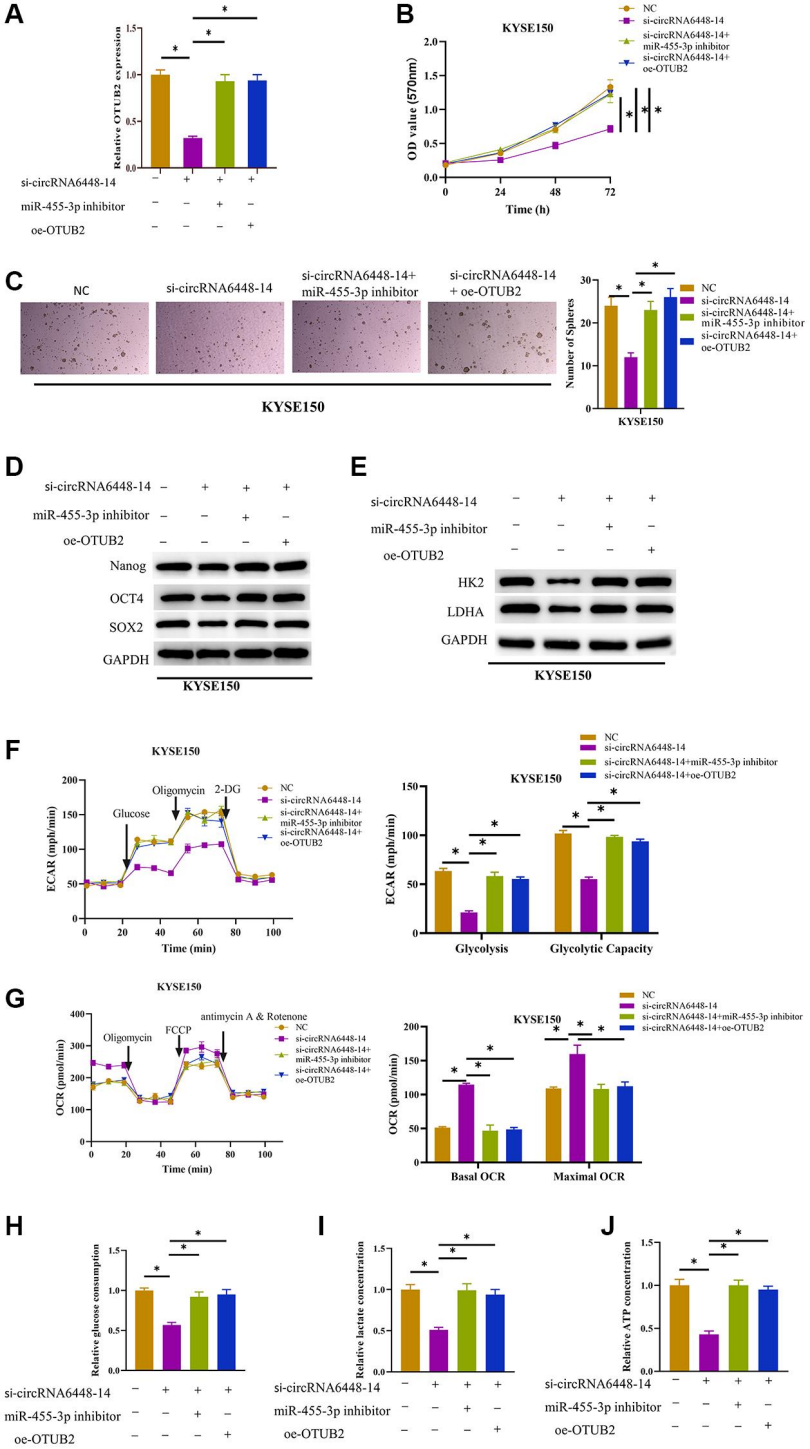
**Stimulative effect on cell glycolysis and stemness by circRNA6448-14/miR-455-3p/OTUB2 axis.** (**A**) qRT-PCR detection of OTUB2 expression in different treatment groups. (**B**) MTT detection of cell viability in different treatment groups. (**C**) Stem cell sphere formation assay in different treatment groups. (**D**, **E**) WB detection of stem cell surface marker proteins Nanog, OCT4, SOX2 expression and glycolysis rate-limiting enzymes HK2, LDHA expression in different treatment groups. (**F**, **G**) The ECAR and OCR of different treatment groups. (**H**–**J**) Glucose consumption, lactate production, and ATP generation levels in cells of different treatment groups. ^*^P < 0.05.

After confirming the functional relationship between circRNA6448-14/miR-455-3p/OTUB2 axis, we further investigated the effects of this regulatory axis on ESCC cell glycolysis and their stemness. First, cell groups were constructed for subsequent experiments: NC (si-NC+inhibitor-NC+oe-NC treatment), si-circRNA6448-14 (si-circRNA6448-14+inhibitor-NC+oe-NC treatment), si-circRNA6448-14+miR-455-3p inhibitor (si-circRNA6448-14+miR-455-3p inhibitor+oe-NC treatment), and si-circRNA6448-14+oe-OTUB2 (si-circRNA6448-14+inhibitor-NC+oe-OTUB2 treatment). According to the results, OTUB2 expression in KYSE150 cells was considerably reduced by circRNA6448-14 knockdown alone when compared to the other three groups (Figure 4A). The inhibitory effect of knocking down circRNA6448-14 on cell viability was eliminated by adding miR-455-3p inhibitor or overexpressing OTUB2 (Figure 4B). The stem cell sphere formation assay demonstrated that the inhibitory effect on sphere formation mediated by circRNA6448-14 knockdown could be reversed by inhibiting miR-455-3p or overexpressing OTUB2 (Figure 4C). The expression of cell surface marker proteins and glycolysis rate-limiting enzyme was significantly reduced in the circRNA6448-14 knockdown group and restored upon the addition of miR-455-3p inhibitor or overexpression of OTUB2 (Figure 4D, 4E). Furthermore, knocking down circRNA6448-14 inhibited the ECAR of KYSE150 cells, while inhibiting miR-455-3p or overexpressing OTUB2 restored the ECAR of KYSE150 cells (Figure 4F). In contrast, the knockdown of circRNA6448-14 promoted OCR in KYSE150 cells, and restoration of OCR to control levels was observed by adding miR-455-3p inhibitor or overexpressing OTUB2 (Figure 4G). Finally, the determination of glycolysis phenotype-related indicators showed that the inhibitory effects on lactate production, glucose consumption, and ATP production caused by knocking down circRNA6448-14 could be reversed by miR-455-3p inhibitor or overexpression of OTUB2 (Figure 4H–4J). In conclusion, circRNA6448-14 sponged miR-455-3p, which in turn promoted OTUB2 expression, thereby enhancing glycolysis and stemness of ESCC cells.

## DISCUSSION

Cancer cells reprogrammed their metabolism to become “glycolysis-dominant” which enables them to meet their energy and macromolecule need and enhances their survival chances. It is also called the “Warburg effect”, which gives cancer cells an advantage in survival and increases the carcinogenic potential of the tumor environment [37]. However, multiple mechanisms contributed to the abnormal glycolysis in tumor cells, including abnormal gene expression [38], abnormal RNA modifications [39, 40], and abnormal post-translational modifications [41]. Recently, the function of circRNA in ESCC cell glycolysis has been evident [42]. For example, circGOT1 adsorbs miR-606 to promote the expression of GOT1, thereby inducing ESCC cell migration, proliferation, aerobic glycolysis, and cisplatin resistance [43]. By absorbing miR-497-5p, CircDUSP16 knockdown controls TKTL1 expression and prevents ESCC cell growth, invasion, and glycolysis [44]. In our study, it was found that knocking down circRNA6448-14 could prevent the glycolysis and stemness of ESCC cells. We identified circRNA6448-14 as a novel oncogene in ESCC that could accelerate the tumor growth by triggering a reprogramming of glycolysis.

In addition, cancer “stemness” is the basis for the existence of cancer, which defines the ability of cancer cells to persist and differentiate indefinitely [45], however, the regulation of cancer cell stemness may also depend on circRNA [46], and circSLC7A11 knockdown can reduce stemness of laryngeal squamous cell carcinoma cells [47]. hsa_circ_0001741 was proved to act as a miRNA sponge and prevent miR-491-5p from inhibiting ESCC cell stemness [48]. In line with earlier research, we discovered that circRNA6448-14 knockdown greatly suppressed the stemness marker Nanog, OCT4, SOX2 in ESCC. Nanog, OCT4, SOX2 are recognized as key stem cell regulators that maintain the ability of stem cells to self-renew, proliferate and differentiate, transforming cancer cells into a stem cell-like phenotype [49–51]. These results demonstrated the importance of circRNA6448-14 as a therapeutic target for ESCC.

circRNA6448-14 had been confirmed to serve as a sponge for miR-455-3 [28]. Through relevant experiments, this study further validated the binding relationship between miR-455-3p and circRNA6448-14. We used miRwalk to predict the downstream target gene (OTUB2) of miR-455-3p and obtained a complete circRNA-miRNA-mRNA regulatory network. The molecular structure of OTUB2 consists of a central five-stranded β-sheet, flanking regions of small helices at the amino terminus (α1, α2), and a large helical region (α3-α8). It is a deubiquitinating enzyme [52]. Zhang et al. [53] measured cancer metastasis by *in vivo* screening system and demonstrated that OTUB2 is the most indispensable gene that induces metastasis among the screened deubiquitinating enzymes. OTUB2 could make a binding relationship and deubiquitination, thereby stabilizing YAP and TAZ to activate the Hippo pathway, stimulate cancer stem cell characteristics, and maintain tumor cell proliferation and metastasis. Similarly, in non-small cell lung cancer, OTUB2 could bind to U2AF2 and deubiquitinate it, making U2AF2 more stable, promoting the Warburg effect of tumors through the AKT/mTOR signaling pathway [54]. Surprisingly, opposite results were observed in ESCC. Liu et al. [55] believed that OTUB2 is overexpressed in ESCC and can directly stabilize and interact with YAP1/TAZ through SUMO, leading to the stimulation of YAP1/TAZ expression and activation of downstream target genes such as CTGF and CYR61, thereby promoting tumor cell progression. Our results also indicated a high expression of OTUB2 in ESCC. In contrast, Chang et al. [56] discovered that OTUB2 is missing in ESCC tissues, while OTUB2 can deubiquitinate STAT1 and phosphorylate it, thereby activating CALML3 transcription and phospholipid acetylserine synthesis to exert tumor suppressive effects. The dual role of OTUB2 in cancer may be the result of its interaction with different proteins as a deubiquitinating enzyme. Deubiquitination is well known to contribute to the stability of proteins [57, 58]. OTUB2 stimulates the growth of cancer by acting on oncogenic proteins. It has a suppressive effect when it interacts with tumor suppressor proteins, which causes ESCC to react in an entirely different way. Finally, we revealed that circRNA6448-14 promoted OTUB2 expression and enhanced ESCC glycolysis and stemness by competitively binding to miR-455-3p. This is similar to the regulatory mechanism of circRNA-miRNA-mRNA network on glycolysis and stemness in hepatocellular carcinoma reported by Feng et al. [59]. Our findings make a substantial contribution to the understanding of the comparatively sparse circRNA-miRNA-mRNA regulatory network in ESCC.

In summary, this study demonstrated that by competitively binding with miR-455-3p, circRNA6448-14 enhanced OTUB2 expression, ESCC glycolysis, and stemness. Currently, the significance of the circRNA-miRNA-mRNA network in ESCC is still unclear, and our data provided a new circRNA-miRNA-mRNA regulatory network for the ESCC pathogenesis. However, it is unknown whether this regulatory axis plays a carcinogenic role in ESCC by affecting glycolysis and stemness only and has an impact on other pathways or metabolism. Also, this study lacked exploration at clinical and animal levels, but only validated at the cellular level, which was a limitation of this study. We would investigate additional potential effects of this regulatory axis in the upcoming research, which would aid in the creation of novel therapy for ESCC patients.

## References

[1] Sung H, Ferlay J, Siegel RL, Laversanne M, Soerjomataram I, Jemal A, Bray F. Global Cancer Statistics 2020: GLOBOCAN Estimates of Incidence and Mortality Worldwide for 36 Cancers in 185 Countries. CA Cancer J Clin. 2021; 71:209–49. 10.3322/caac.21660 33538338

[2] Morgan E, Soerjomataram I, Rumgay H, Coleman HG, Thrift AP, Vignat J, Laversanne M, Ferlay J, Arnold M. The Global Landscape of Esophageal Squamous Cell Carcinoma and Esophageal Adenocarcinoma Incidence and Mortality in 2020 and Projections to 2040: New Estimates From GLOBOCAN 2020. Gastroenterology. 2022; 163:649–58.e2. 10.1053/j.gastro.2022.05.054 35671803

[3] Lagergren J, Smyth E, Cunningham D, Lagergren P. Oesophageal cancer. Lancet. 2017; 390:2383–96. 10.1016/S0140-6736(17)31462-9 28648400

[4] Li M, Park JY, Sheikh M, Kayamba V, Rumgay H, Jenab M, Narh CT, Abedi-Ardekani B, Morgan E, de Martel C, McCormack V, Arnold M. Population-based investigation of common and deviating patterns of gastric cancer and oesophageal cancer incidence across populations and time. Gut. 2023; 72:846–54. 10.1136/gutjnl-2022-328233 36241389

[5] Matsueda K, Ishihara R. Preoperative Diagnosis and Indications for Endoscopic Resection of Superficial Esophageal Squamous Cell Carcinoma. J Clin Med. 2020; 10:13. 10.3390/jcm10010013 33374639 PMC7793475

[6] Yamamoto S, Kato K. JUPITER-06 establishes immune checkpoint inhibitors as essential first-line drugs for the treatment of advanced esophageal squamous cell carcinoma. Cancer Cell. 2022; 40:238–40. 10.1016/j.ccell.2022.02.009 35245448

[7] Barbato L, Bocchetti M, Di Biase A, Regad T. Cancer Stem Cells and Targeting Strategies. Cells. 2019; 8:926. 10.3390/cells8080926 31426611 PMC6721823

[8] Taniguchi D, Saeki H, Nakashima Y, Kudou K, Nakanishi R, Kubo N, Ando K, Oki E, Oda Y, Maehara Y. CD44v9 is associated with epithelial-mesenchymal transition and poor outcomes in esophageal squamous cell carcinoma. Cancer Med. 2018; 7:6258–68. 10.1002/cam4.1874 30474922 PMC6308082

[9] Clara JA, Monge C, Yang Y, Takebe N. Targeting signalling pathways and the immune microenvironment of cancer stem cells - a clinical update. Nat Rev Clin Oncol. 2020; 17:204–32. 10.1038/s41571-019-0293-2 31792354

[10] Chen LL. The expanding regulatory mechanisms and cellular functions of circular RNAs. Nat Rev Mol Cell Biol. 2020; 21:475–90. 10.1038/s41580-020-0243-y 32366901

[11] Meng F, Zhang X, Wang Y, Lin J, Tang Y, Zhang G, Qiu B, Zeng X, Liu W, He X. Hsa_circ_0021727 (circ-CD44) promotes ESCC progression by targeting miR-23b-5p to activate the TAB1/NFκB pathway. Cell Death Dis. 2023; 14:9. 10.1038/s41419-022-05541-x 36609391 PMC9822936

[12] Wang J, Yue BL, Huang YZ, Lan XY, Liu WJ, Chen H. Exosomal RNAs: Novel Potential Biomarkers for Diseases-A Review. Int J Mol Sci. 2022; 23:2461. 10.3390/ijms23052461 35269604 PMC8910301

[13] Min X, Liu DL, Xiong XD. Circular RNAs as Competing Endogenous RNAs in Cardiovascular and Cerebrovascular Diseases: Molecular Mechanisms and Clinical Implications. Front Cardiovasc Med. 2021; 8:682357. 10.3389/fcvm.2021.682357 34307497 PMC8292644

[14] Patil NS, Feng B, Su Z, Castellani CA, Chakrabarti S. Circular RNA mediated gene regulation in chronic diabetic complications. Sci Rep. 2021; 11:23766. 10.1038/s41598-021-02980-y 34887449 PMC8660871

[15] Shan C, Zhang Y, Hao X, Gao J, Chen X, Wang K. Biogenesis, functions and clinical significance of circRNAs in gastric cancer. Mol Cancer. 2019; 18:136. 10.1186/s12943-019-1069-0 31519189 PMC6743094

[16] Li X, Wang J, Zhang C, Lin C, Zhang J, Zhang W, Zhang W, Lu Y, Zheng L, Li X. Circular RNA circITGA7 inhibits colorectal cancer growth and metastasis by modulating the Ras pathway and upregulating transcription of its host gene ITGA7. J Pathol. 2018; 246:166–79. 10.1002/path.5125 29943828

[17] Di X, Jin X, Li R, Zhao M, Wang K. CircRNAs and lung cancer: Biomarkers and master regulators. Life Sci. 2019; 220:177–85. 10.1016/j.lfs.2019.01.055 30711537

[18] Zhao CH, Qu L, Zhang H, Qu R. Identification of breast cancer-related circRNAs by analysis of microarray and RNA-sequencing data: An observational study. Medicine (Baltimore). 2019; 98:e18042. 10.1097/MD.0000000000018042 31725681 PMC6867785

[19] Patop IL, Wüst S, Kadener S. Past, present, and future of circRNAs. EMBO J. 2019; 38:e100836. 10.15252/embj.2018100836 31343080 PMC6694216

[20] Fan L, Cao Q, Liu J, Zhang J, Li B. Circular RNA profiling and its potential for esophageal squamous cell cancer diagnosis and prognosis. Mol Cancer. 2019; 18:16. 10.1186/s12943-018-0936-4 30674324 PMC6343327

[21] Gu L, Sang Y, Nan X, Zheng Y, Liu F, Meng L, Sang M, Shan B. circCYP24A1 facilitates esophageal squamous cell carcinoma progression through binding PKM2 to regulate NF-κB-induced CCL5 secretion. Mol Cancer. 2022; 21:217. 10.1186/s12943-022-01686-7 36514094 PMC9746112

[22] Hu X, Wu D, He X, Zhao H, He Z, Lin J, Wang K, Wang W, Pan Z, Lin H, Wang M. circGSK3β promotes metastasis in esophageal squamous cell carcinoma by augmenting β-catenin signaling. Mol Cancer. 2019; 18:160. 10.1186/s12943-019-1095-y 31722716 PMC6854808

[23] Li J, Song Y, Cai H, Zhou B, Ma J. Roles of circRNA dysregulation in esophageal squamous cell carcinoma tumor microenvironment. Front Oncol. 2023; 13:1153207. 10.3389/fonc.2023.1153207 37384299 PMC10299836

[24] Shen Y, Shao Y, Niu C, Ruan X, Zang Z, Nakyeyune R, Guo X, Liu F. Systematic Identification of circRNA-miRNA-mRNA Regulatory Network in Esophageal Squamous Cell Carcinoma. Front Genet. 2021; 12:580390. 10.3389/fgene.2021.580390 33747034 PMC7966720

[25] Wang C, Zhou M, Zhu P, Ju C, Sheng J, Du D, Wan J, Yin H, Xing Y, Li H, He J, He F. IGF2BP2-induced circRUNX1 facilitates the growth and metastasis of esophageal squamous cell carcinoma through miR-449b-5p/FOXP3 axis. J Exp Clin Cancer Res. 2022; 41:347. 10.1186/s13046-022-02550-8 36522683 PMC9753396

[26] Zhou Q, Lei C, Cui F, Chen H, Cao X. Circ-ATIC regulates esophageal squamous cell carcinoma growth and metastasis through miR-1294/PBX3 pathway. Heliyon. 2023; 9:e12916. 10.1016/j.heliyon.2023.e12916 36699282 PMC9868444

[27] Song D, Ye Z, Chen F, Zhan L, Sun X. circFNDC3B promotes esophageal squamous cell carcinoma progression by targeting MYO5A via miR-370-3p/miR-136-5p. BMC Cancer. 2023; 23:821. 10.1186/s12885-023-11314-2 37667251 PMC10476377

[28] Zhang Y, Yuan X, Yue N, Wang L, Liu J, Dai N, Yang H, Fan R, Zhou F. hsa_circRNA6448-14 promotes carcinogenesis in esophageal squamous cell carcinoma. Aging (Albany NY). 2020; 12:15581–602. 10.18632/aging.103650 32805720 PMC7467364

[29] Zhao R, Chen P, Qu C, Liang J, Cheng Y, Sun Z, Tian H. Circular RNA circTRPS1-2 inhibits the proliferation and migration of esophageal squamous cell carcinoma by reducing the production of ribosomes. Cell Death Discov. 2023; 9:5. 10.1038/s41420-023-01300-9 36635258 PMC9837173

[30] Wang X, Liu Z, Du Y, Hao S, Zhao B. Hsa_circ_0043603 promotes the progression of esophageal squamous cell carcinoma by sponging miR-1178-3p and regulating AADAC expression. Heliyon. 2023; 9:e19807. 10.1016/j.heliyon.2023.e19807 37809396 PMC10559168

[31] Feng Z, Wu J. hsa_circ_0129047 Upregulates LYVE1 to Inhibit Hepatocellular Carcinoma Progression by Sponging miR-492. Dis Markers. 2023; 2023:6978234. 10.1155/2023/6978234 37810197 PMC10560114

[32] Zhao Y, Song J, Dong W, Liu X, Yang C, Wang D, Xue Y, Ruan X, Liu L, Wang P, Zhang M, Liu Y. The MBNL1/circNTRK2/PAX5 pathway regulates aerobic glycolysis in glioblastoma cells by encoding a novel protein NTRK2-243aa. Cell Death Dis. 2022; 13:767. 10.1038/s41419-022-05219-4 36064939 PMC9445070

[33] Fan Q, Lu Q, Wang G, Zhu W, Teng L, Chen W, Bi L. Optimizing component formula suppresses lung cancer by blocking DTL-mediated PDCD4 ubiquitination to regulate the MAPK/JNK pathway. J Ethnopharmacol. 2022; 299:115546. 10.1016/j.jep.2022.115546 35850313

[34] Tang Q, Chen J, Di Z, Yuan W, Zhou Z, Liu Z, Han S, Liu Y, Ying G, Shu X, Di M. TM4SF1 promotes EMT and cancer stemness via the Wnt/β-catenin/SOX2 pathway in colorectal cancer. J Exp Clin Cancer Res. 2020; 39:232. 10.1186/s13046-020-01690-z 33153498 PMC7643364

[35] Li L, Liang Y, Kang L, Liu Y, Gao S, Chen S, Li Y, You W, Dong Q, Hong T, Yan Z, Jin S, Wang T, et al. Transcriptional Regulation of the Warburg Effect in Cancer by SIX1. Cancer Cell. 2018; 33:368–85.e7. 10.1016/j.ccell.2018.01.010 29455928

[36] Kristensen LS, Andersen MS, Stagsted LVW, Ebbesen KK, Hansen TB, Kjems J. The biogenesis, biology and characterization of circular RNAs. Nat Rev Genet. 2019; 20:675–91. 10.1038/s41576-019-0158-7 31395983

[37] Chelakkot C, Chelakkot VS, Shin Y, Song K. Modulating Glycolysis to Improve Cancer Therapy. Int J Mol Sci. 2023; 24:2606. 10.3390/ijms24032606 36768924 PMC9916680

[38] Jiang H, Wei H, Wang H, Wang Z, Li J, Ou Y, Xiao X, Wang W, Chang A, Sun W, Zhao L, Yang S. Zeb1-induced metabolic reprogramming of glycolysis is essential for macrophage polarization in breast cancer. Cell Death Dis. 2022; 13:206. 10.1038/s41419-022-04632-z 35246504 PMC8897397

[39] Wu Y, Chen Z, Xie G, Zhang H, Wang Z, Zhou J, Chen F, Li J, Chen L, Niu H, Wang H. RNA m^1^A methylation regulates glycolysis of cancer cells through modulating ATP5D. Proc Natl Acad Sci U S A. 2022; 119:e2119038119. 10.1073/pnas.2119038119 35867754 PMC9282374

[40] Li Z, Peng Y, Li J, Chen Z, Chen F, Tu J, Lin S, Wang H. N^6^-methyladenosine regulates glycolysis of cancer cells through PDK4. Nat Commun. 2020; 11:2578. 10.1038/s41467-020-16306-5 32444598 PMC7244544

[41] Zhang Z, Li X, Yang F, Chen C, Liu P, Ren Y, Sun P, Wang Z, You Y, Zeng YX, Li X. DHHC9-mediated GLUT1 S-palmitoylation promotes glioblastoma glycolysis and tumorigenesis. Nat Commun. 2021; 12:5872. 10.1038/s41467-021-26180-4 34620861 PMC8497546

[42] Yu T, Wang Y, Fan Y, Fang N, Wang T, Xu T, Shu Y. CircRNAs in cancer metabolism: a review. J Hematol Oncol. 2019; 12:90. 10.1186/s13045-019-0776-8 31484561 PMC6727394

[43] Zhou S, Guo Z, Lv X, Zhang X. CircGOT1 promotes cell proliferation, mobility, and glycolysis-mediated cisplatin resistance via inhibiting its host gene GOT1 in esophageal squamous cell cancer. Cell Cycle. 2022; 21:247–60. 10.1080/15384101.2021.2015671 34919012 PMC8855861

[44] Ma L, Li H, Lin Y, Wang G, Xu Q, Chen Y, Xiao K, Rao X. CircDUSP16 Contributes to Cell Development in Esophageal Squamous Cell Carcinoma by Regulating miR-497-5p/TKTL1 Axis. J Surg Res. 2021; 260:64–75. 10.1016/j.jss.2020.11.052 33326930

[45] Alsayed RKM, Sheikhan KSA, Alam MA, Buddenkotte J, Steinhoff M, Uddin S, Ahmad A. Epigenetic programing of cancer stemness by transcription factors-non-coding RNAs interactions. Semin Cancer Biol. 2023; 92:74–83. 10.1016/j.semcancer.2023.04.005 37054905

[46] Lin Z, Tang X, Wan J, Zhang X, Liu C, Liu T. Functions and mechanisms of circular RNAs in regulating stem cell differentiation. RNA Biol. 2021; 18:2136–49. 10.1080/15476286.2021.1913551 33896374 PMC8632079

[47] Yu W, He X, Zhang C, Huang F. Circular RNA circSLC7A11 contributes to progression and stemness of laryngeal squamous cell carcinoma via sponging miR-877-5p from LASP1. Heliyon. 2023; 9:e18290. 10.1016/j.heliyon.2023.e18290 37539185 PMC10393633

[48] Li L, Lei K, Lyu Y, Tan B, Liang R, Wu D, Wang K, Wang W, Lin H, Wang M. hsa_circ_0001741 promotes esophageal squamous cell carcinoma stemness, invasion and migration by sponging miR-491-5p to upregulate NOTCH3 expression. Am J Cancer Res. 2022; 12:2012–31. 35693080 PMC9185627

[49] Vasefifar P, Motafakkerazad R, Maleki LA, Najafi S, Ghrobaninezhad F, Najafzadeh B, Alemohammad H, Amini M, Baghbanzadeh A, Baradaran B. Nanog, as a key cancer stem cell marker in tumor progression. Gene. 2022; 827:146448. 10.1016/j.gene.2022.146448 35337852

[50] Mohiuddin IS, Wei SJ, Kang MH. Role of OCT4 in cancer stem-like cells and chemotherapy resistance. Biochim Biophys Acta Mol Basis Dis. 2020; 1866:165432. 10.1016/j.bbadis.2019.03.005 30904611 PMC6754810

[51] Novak D, Hüser L, Elton JJ, Umansky V, Altevogt P, Utikal J. SOX2 in development and cancer biology. Semin Cancer Biol. 2020; 67:74–82. 10.1016/j.semcancer.2019.08.007 31412296

[52] Nanao MH, Tcherniuk SO, Chroboczek J, Dideberg O, Dessen A, Balakirev MY. Crystal structure of human otubain 2. EMBO Rep. 2004; 5:783–8. 10.1038/sj.embor.7400201 15258613 PMC1299112

[53] Zhang Z, Du J, Wang S, Shao L, Jin K, Li F, Wei B, Ding W, Fu P, van Dam H, Wang A, Jin J, Ding C, et al. OTUB2 Promotes Cancer Metastasis via Hippo-Independent Activation of YAP and TAZ. Mol Cell. 2019; 73:7–21.e7. 10.1016/j.molcel.2018.10.030 30472188

[54] Li J, Cheng D, Zhu M, Yu H, Pan Z, Liu L, Geng Q, Pan H, Yan M, Yao M. OTUB2 stabilizes U2AF2 to promote the Warburg effect and tumorigenesis via the AKT/mTOR signaling pathway in non-small cell lung cancer. Theranostics. 2019; 9:179–95. 10.7150/thno.29545 30662561 PMC6332791

[55] Liu L, Cheng H, Ji M, Su L, Lu Z, Hu X, Guan Y, Xiao J, Ma L, Zhang W, Pu H. OTUB2 Regulates YAP1/TAZ to Promotes the Progression of Esophageal Squamous Cell Carcinoma. Biol Proced Online. 2022; 24:10. 10.1186/s12575-022-00169-9 35850645 PMC9290284

[56] Chang W, Luo Q, Wu X, Nan Y, Zhao P, Zhang L, Luo A, Jiao W, Zhu Q, Fu Y, Liu Z. OTUB2 exerts tumor-suppressive roles via STAT1-mediated CALML3 activation and increased phosphatidylserine synthesis. Cell Rep. 2022; 41:111561. 10.1016/j.celrep.2022.111561 36288705

[57] Wu X, Wang H, Zhu D, Chai Y, Wang J, Dai W, Xiao Y, Tang W, Li J, Hong L, Pei M, Zhang J, Lin Z, et al. USP3 promotes gastric cancer progression and metastasis by deubiquitination-dependent COL9A3/COL6A5 stabilisation. Cell Death Dis. 2021; 13:10. 10.1038/s41419-021-04460-7 34930901 PMC8688524

[58] Shi J, Zhang Q, Yin X, Ye J, Gao S, Chen C, Yang Y, Wu B, Fu Y, Zhang H, Wang Z, Wang B, Zhu Y, et al. Stabilization of IGF2BP1 by USP10 promotes breast cancer metastasis via CPT1A in an m6A-dependent manner. Int J Biol Sci. 2023; 19:449–64. 10.7150/ijbs.76798 36632454 PMC9830507

[59] Feng Y, Xia S, Hui J, Xu Y. Circular RNA circBNC2 facilitates glycolysis and stemness of hepatocellular carcinoma through the miR-217/high mobility group AT-hook 2 (HMGA2) axis. Heliyon. 2023; 9:e17120. 10.1016/j.heliyon.2023.e17120 37360090 PMC10285170

